# Assessing head injury risk and neuroprotective effect of ketone monoester supplementation in military airborne training

**DOI:** 10.14814/phy2.70818

**Published:** 2026-03-19

**Authors:** Toshiya Miyatsu, Jeremy McAdam, Zachary A. Graham, Arash Mahyari, Konstantinos Mitsopoulos, Andrew Dorsey, Meredith Yeager, Art Finch, Eric Alsop, Elizabeth Hutchins, Brooke Lovell, Krystine Garcia‐Mansfield, Bessie Meechoovet, Joanna Palada, Ritin Sharma, Kendall Van Keuren‐Jensen, Patrick Pirrotte, Marcas Bamman, Timothy Broderick, Morley Stone

**Affiliations:** ^1^ Healthspan, Resilience and Performance Research, Florida Institute for Human and Machine Cognition Pensacola Florida USA; ^2^ Bioinnovation and Genome Sciences Division Translational Genomics Research Institute Phoenix Arizona USA; ^3^ Early Detection and Prevention Division Translational Genomics Research Institute Phoenix Arizona USA; ^4^ Integrated Mass Spectrometry Shared Resource, City of Hope National Medical Center Duarte California USA

**Keywords:** ketone monoester supplementation, mild traumatic brain injury, military airborne training, neuroprotection, subconcussive head impact

## Abstract

Warfighters face significant risk of mild traumatic brain injury (mTBI) during operations and training. We performed a double‐blind, randomized, placebo‐controlled trial to: (1) determine head strike (HS) incidence, during US Army Basic Airborne Course's Improved Swing Landing Trainer (ISLT) training, (2) test protective efficacy of ketone monoester (KME) supplementation, and (3) assess blood‐based mTBI biomarker signatures. We enrolled 354 active‐duty male and female service members randomized 1:1 to placebo (PLA) or KME; *n* = 318 completed ISLT and assessments (PLA = 157, 142 M/15F; KME = 161, 144 M/18F). *N* = 112 (35.2%; 35.7% in PLA, 34.8% in KME) experienced HS, defined as whiplash or helmet‐to‐ground impact. Assessments included the Automated Neuropsychological Assessment Metric (ANAM‐4), SWAY balance testing, and plasma biomarkers. One ANAM‐4 measure (Simple Reaction Time‐Repeat; *p* = 0.024) and one SWAY measure (Single Leg Right; *p* = 0.014) demonstrated significant HS × Time × Treatment interactions, with performance decrements in HS + PLA not observed in HS + KME. Several HS × Time interactions were observed for ANAM‐4 and SWAY variables, indicating HS‐induced cognitive and balance decline. No relevant blood‐based biomarker patterns were associated with HS or KME. Predictive modeling identified variables associated with HS risk with moderate accuracy. In conclusion, ISLT resulted in ~32% head injury incidence and KME supplementation showed limited protection of cognitive and balance without corresponding biomarker changes.

## 1 INTRODUCTION

Traumatic brain injury (TBI) significantly affects warfighters. US military personnel deployed to Iraq and Afghanistan had prevalence rates of TBI ranging from 11% to 23% (Lindquist et al., 2017). However, mild traumatic brain injury (mTBI) that occurs during operations or training may not be readily identified and immediately treated due to late presentation, heterogeneity, and subtlety of symptoms. Recognizing the importance of early detection and treatment, the Department of Defense (DoD) and Department of Veterans Affairs (VA) have established system‐wide screening and assessment procedures to identify concussion in service members and veterans at the earliest opportunity (Defense and Veteran Brain Injury Center, 2020). Improved mTBI prevention, diagnosis, and treatment remain high priority areas for military medical research (Lee et al., 2020; Turgoose & Murphy, 2018).

Studies of US Army airborne operations and training have identified the incidence of moderate to severe closed head injury to be relatively infrequent (Army Public Health Service, 2015; Ivins et al., 2008; Knapik et al., 2011, 2014a, 2014b). However, repetitive, low‐magnitude head accelerations and impacts that do not produce clinical symptoms, termed subconcussive head impacts (SHI), occur frequently in activities such as parachute landing, combatives, and contact sports (Kelley et al., 2021; Slobounov et al., 2017). These events are recognized as a distinct category of neurological insult characterized by transient physiological stress without overt concussion diagnosis. While repetitive SHI have been implicated in long‐term neurodegenerative conditions such as chronic traumatic encephalopathy, there is currently limited evidence that airborne training confers greater chronic neurological risk than other military roles, with most long‐term outcome data derived from blast‐exposed or contact‐combat populations (Jordan, 2013; McKee et al., 2013; Stein et al., 2014). Even in the absence of symptoms, SHI have been shown to transiently impair cognitive performance (e.g., slowed reaction time and reduced executive control), alter balance and postural stability measures, and induce acute changes in neuro‐axonal and astroglial blood biomarkers such as GFAP, UCH‐L1, and NFL (Davenport et al., 2016; Kelley et al., 2021). Repetitive SHI exposure has been linked to cumulative neurophysiological changes and possible long‐term neurodegeneration risk (Koerte et al., 2015). Despite these emerging findings, SHI are rarely monitored in operational military environments, leaving an important knowledge gap in understanding their short‐term functional and physiological consequences.

Current standard‐of‐care for head impact injury, whether concussive or subconcussive, remains largely supportive and does not target the secondary cellular energy crisis, oxidative stress, and inflammation that follow mechanical insult (Jarrahi et al., 2020). With the recognized risk of SHI during airborne operations, prophylactic administration of a safe and well‐tolerated therapeutic could reduce secondary neuronal injury following head impact and mitigate the negative long‐term impact on warfighter health and performance. Acute or prophylactic treatment capable of stabilizing neuronal metabolism and preventing secondary injury therefore represents a critical unmet need (Diaz‐Arrastia et al., 2014).

Therapeutic ketosis, defined as a physiological state where blood ketone levels are >0.5 mM, has demonstrated improved structural and functional outcomes in the setting of neurological injury, including TBI (Prins & Matsumoto, 2014; White & Venkatesh, 2011). The underlying pathophysiology of TBI involves a transient increase in brain glucose metabolism followed by a prolonged depression of brain glucose metabolism, during which there is increased free radical production and subsequent DNA damage (White & Venkatesh, 2011). Given the need for a readily available energy substrate and the impairment of glucose metabolism following TBI, ketones have been demonstrated to provide a more efficacious source of energy to the brain following injury (White & Venkatesh, 2011). Ketones are more energy efficient than glucose and protect against apoptosis and necrosis by attenuating the formation of reactive oxygen species and increasing antioxidant capacity (Kim et al., 2007; Shimazu et al., 2013; Youm et al., 2015). Through these mechanisms, exogenous ketone monoester (KME) supplementation may preserve neuronal function and synaptic signaling during the post‐impact metabolic depression that underlies cognitive and balance performance decrements after SHI.

In contrast to the slow and variable process of inducing ketosis through fasting or a ketogenic diet (KD), transient ketosis can be induced using generally recognized as safe (GRAS) ketone dietary supplements. Ketone monoester (KME) supplements provide rapid (<30‐min) and sustained (~5‐h) ketosis in a predictable and dose‐dependent manner (Evans et al., 2017; Shivva et al., 2016; Stubbs et al., 2020). KME, which are primarily composed of beta‐hydroxybutyrate and acetoacetate in a fixed ratio, have been well‐studied in healthy adults at rest (Evans et al., 2017; Shivva et al., 2016; Stubbs et al., 2017) and during and after exercise (Cox et al., 2016; Holdsworth et al., 2017; Vandoorne et al., 2017). While therapeutic or dietary ketosis has demonstrated benefit in preclinical models of moderate and severe TBI, its potential to mitigate subtle functional impairments caused by SHI remains unknown. We sought to determine if KME could attenuate head impact‐induced changes in cognitive and balance performance in active‐duty service members going through US Army basic airborne jump training.

Alongside cognitive and balance testing, we assessed a panel of targeted blood‐based biomarkers that have been validated or proposed as indicators of neuronal injury. Glial fibrillary acidic protein (GFAP) and ubiquitin C‐terminal hydrolase‐L1 (UCH‐L1) are FDA‐cleared for aiding in mTBI evaluation (Bazarian et al., 2021; Papa et al., 2024), while neurofilament light (NfL) has strong evidence as a marker of neuro‐axonal injury (Khalil et al., 2018). Homocysteine has also been linked to poorer outcomes after brain injury (Amini et al., 2022; Lauretta et al., 2022). We further measured circulating hormones and cytokines to capture stress, endocrine, and immune responses to head impacts, consistent with evidence for neuroinflammatory and systemic physiological changes following TBI (Corps et al., 2015; Xiong et al., 2018; Zetterberg & Blennow, 2016).

In addition to these targeted assays, extracellular vesicle (EV) transcriptomic and proteomic profiling was performed as an exploratory analysis. EV cargo reflects dynamic cellular responses to injury and has been proposed as a sensitive modality for detecting subclinical neurotrauma (Dey et al., 2023; Hsi et al., 2025; Romero‐García et al., 2025). We therefore included EV transcriptome and proteome analyses to complement targeted biomarker measurements and to generate hypotheses about the biological impact of sub‐concussive headstrikes (HS) and potential modulation by KME.

In summary, our primary objectives were to: (1) quantify HS incidence during US Army Basic Airborne Course training, (2) determine whether HS was associated with acute changes in cognitive and balance performance, and (3) test whether prophylactic KME supplementation could attenuate these HS‐related decrements. Secondary objectives included examining associations with targeted blood biomarkers of neuronal injury, endocrine status, and inflammation. Exploratory aims included multi‐omics profiling and predictive modeling to identify potential molecular signatures and risk factors for HS.

## 2 METHODS

### 2.1 Participants

**FIGURE 1:**
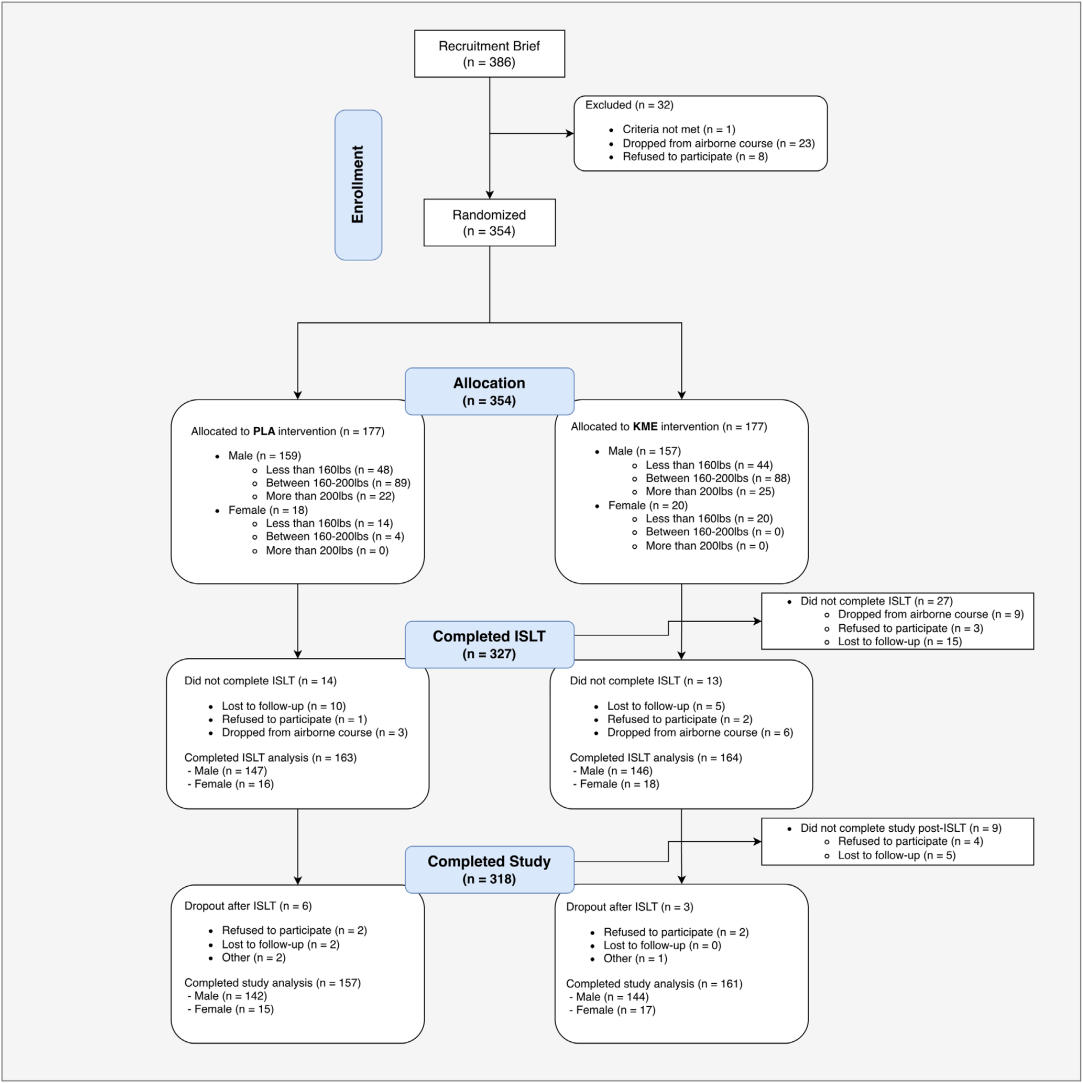
CONSORT diagram.

Active‐duty service members were recruited during US Army Basic Airborne Course (BAC) training at Ft. Moore in Columbus, GA from January 2023 to November 2023. BAC is the largest paratrooper training program in the nation, training around 15,000 students each year. The course is divided into three phases: Ground Week, Tower Week, and Jump Week. Ground Week focuses on the fundamentals of parachuting, physical conditioning, and basic jump techniques. Tower Week builds on these skills with simulated jumps from elevated platforms on towers. In the final phase, Jump Week completes training with a series of parachute jumps from aircraft. Participants were recruited on the Friday before the start of their 3‐week BAC training. They were briefed as a group, given opportunities to ask questions, and if interested in participating were provided with written consent to be a part of the study. Written informed consent was obtained in person by the principal investigator or trained study personnel prior to any study procedures. Participants were informed of their right to decline or withdraw without penalty, and consent was obtained outside the presence of senior active‐duty personnel. The study protocol received approval from the IHMC IRB (2022‐0031), as well as the Office of Human Research Oversight at the US Army Medical Research and Development Command. All procedures were conducted in compliance with local laws and institutional regulations as well as the Declaration of Helsinki. Three hundred and fifty‐four participants enrolled in the study across eight different cohorts, with randomization and group breakdown presented in the CONSORT diagram (Figure 1) and demographics and baseline information presented in Table 1. The cohorts were relatively homogeneous with no independent cohort driving a significant amount of variation in the data.

**TABLE 1:** Demographic characteristics of participants.

	PLA		KME		Total	
	Female	Male	Female	Male	Female	Male
	(*N* = 15)	(*N* = 142)	(*N* = 17)	(*N* = 144)	(*N* = 32)	(*N* = 286)
Education*						
Some high school	0 (0%)	1 (0.7%)	1 (5.9%)	3 (2.1%)	1 (3.1%)	4 (1.4%)
High school/GED	9 (60.0%)	88 (62.0%)	5 (29.4%)	72 (50.0%)	14 (43.8%)	160 (55.9%)
1–3 years after high school	4 (26.7%)	39 (27.5%)	4 (23.5%)	35 (24.3%)	8 (25.0%)	74 (25.9%)
Undergraduate	2 (13.3%)	11 (7.7%)	5 (29.4%)	24 (16.7%)	7 (21.9%)	35 (12.2%)
Advanced degree	0 (0%)	3 (2.1%)	2 (11.8%)	9 (6.3%)	2 (6.3%)	12 (4.2%)
Age (yrs)						
Mean (SD)	24.3 (6.14)	23.6 (4.35)	25.2 (5.21)	24.6 (5.11)	24.8 (5.59)	24.1 (4.77)
Med [Min, Max]	21.0 [19.0, 35.0]	22.0 [18.0, 43.0]	25.0 [19.0, 37.0]	23.0 [18.0, 40.0]	23.0 [19.0, 37.0]	23.0 [18.0, 43.0]
Height (cm)						
Mean (SD)	161 (11.5)	176 (11.6)	160 (9.36)	176 (10.5)	161 (10.2)	176 (11.0)
Med [Min, Max]	165 [132, 175]	178 [123, 200]	160 [145, 183]	178 [121, 200]	161 [132, 183]	178 [121, 200]
Weight (kg)						
Mean (SD)	65.8 (7.37)	79.8 (10.8)	62.9 (6.43)	81.5 (11.6)	64.3 (6.93)	80.6 (11.2)
Med [Min, Max]	68.0 [48.1, 77.1]	79.2 [56.7, 107]	61.2 [54.4, 72.6]	81.6 [54.4, 120]	67.6 [48.1, 77.1]	79.9 [54.4, 120]
BMI						
Mean (SD)	25.5 (3.61)	25.9 (4.43)	24.7 (3.07)	26.4 (3.79)	25.1 (3.30)	26.2 (4.12)
Med [Min, Max]	25.0 [20.8, 35.4]	25.5 [15.9, 53.9]	24.9 [19.9, 32.2]	26.1 [18.3, 44.6]	25.0 [19.9, 35.4]	25.8 [15.9, 53.9]

Note: Continuous variables were compared between PLA and KME within sex using independent‐sample t‐tests; no significant differences were observed. Education level distributions were compared using 𝜒^2^ tests within sex.

Abbreviations: BMI, body mass index; cm, centimeters; kg, kilograms; KME, Ketone Monoester; PLA, Placebo; yrs, years.

* Indicates a significant difference in education distribution between KME and PLA males (𝜒^2^ *p* = 0.031; Fisher's exact *p* = 0.026).

### 2.2 Design

We employed a double‐blind, placebo‐controlled clinical trial in which enrolled participants were randomized 1:1 to receive either KME or placebo (PLA) to determine if supplementation could attenuate head impact induced changes in cognitive and balance performance. The trial and design were built around Improved Swing Landing Trainer (ISLT) training during Tower Week. ISLT training simulates the experience of landing during a parachute jump, with the goal of teaching warfighters how to safely absorb ground impact associated with landing. During ISLT training, students in harnesses jump from a platform approximately 6 feet above the ground and swing forward. After a few back‐and‐forth swings, an instructor who is standing by the suspended student pulls a rope, and the student falls to the ground. The instructor observes whether the student properly executes a Parachute Landing Fall (PLF). Instructor scoring of each student jump also includes HS (whiplash and head impact). Students are required to successfully complete 8 simulated jumps to pass ISLT training.

**FIGURE 2:**
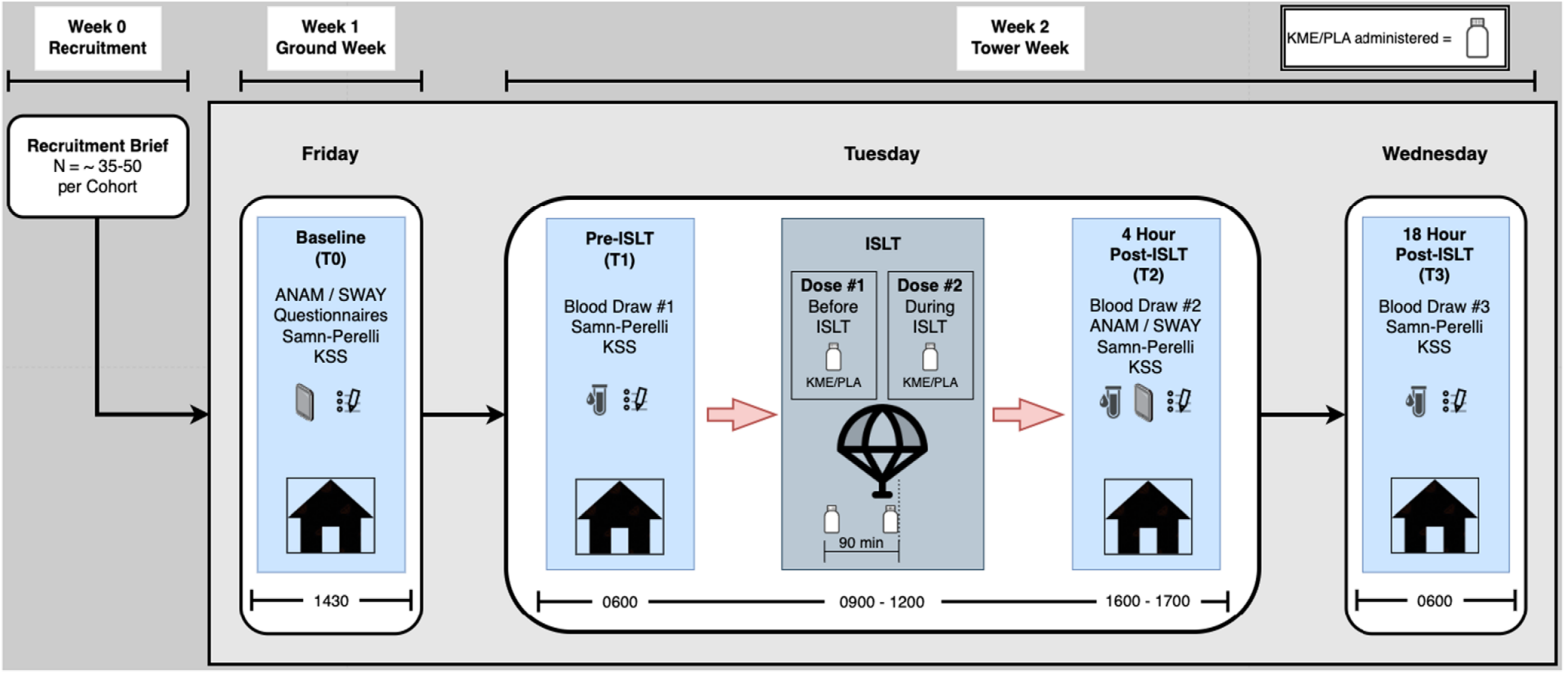
Schematic illustration of data collection schedule.

**FIGURE 3:**
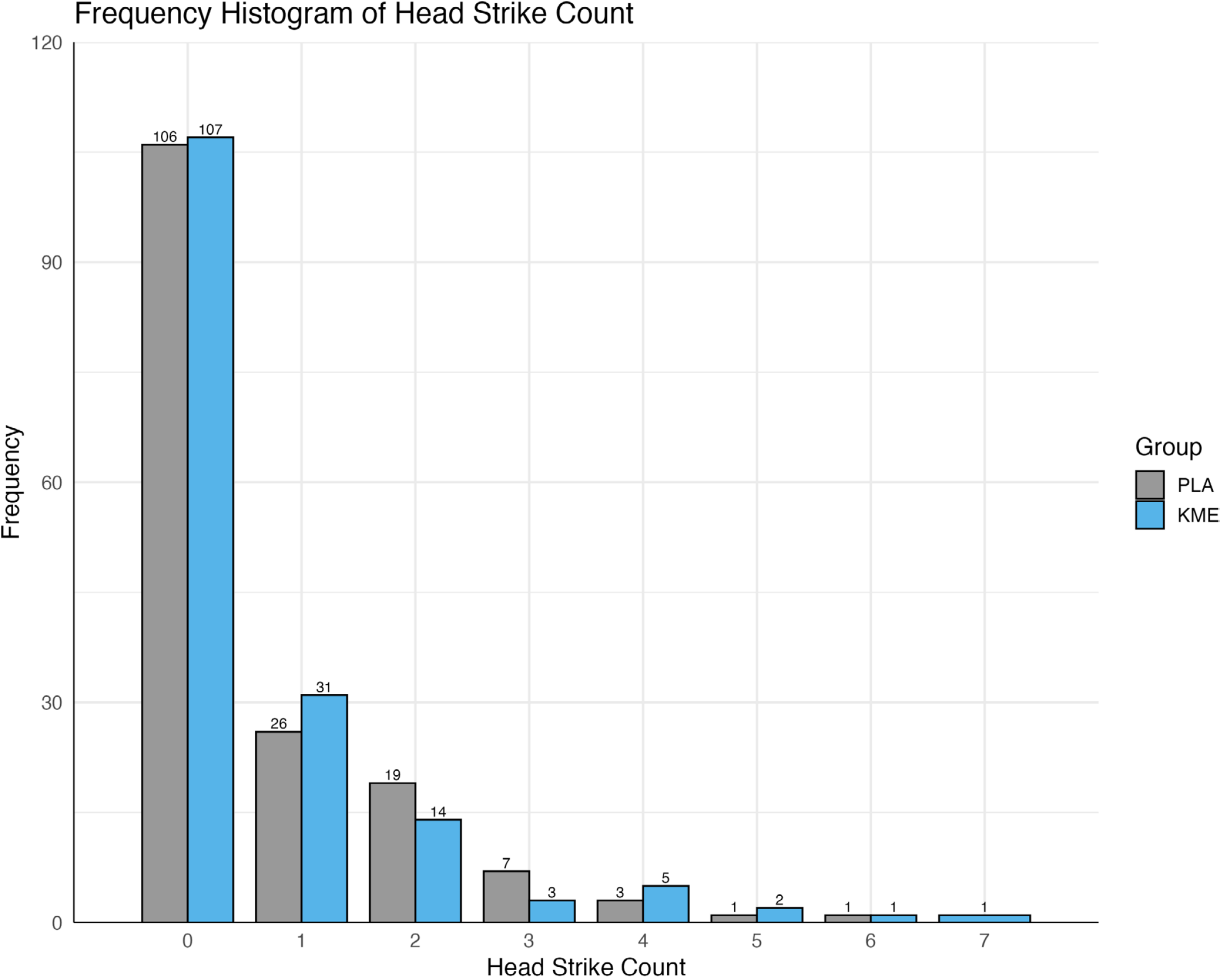
Frequency histogram of the number of headstrikes participants experienced broken down by the treatment group.

Study data collection included 5 sessions during 3 different days: baseline (also referred to as T0), pre‐ISLT (T1), ISLT, ~4 h post‐ISLT (T2), and ~18 h post‐ISLT (T3). The ~4 h and ~18 h post‐ISLT timepoints were selected based on evidence that several plasma biomarkers of mild TBI, such as GFAP and UCH‐L1, peak within 3–6 h post‐injury, whereas others including NFL and certain inflammatory cytokines peak at later (12–24‐h) intervals (Amoo et al., 2022; Bazarian et al., 2021). A schematic illustration of the data collection schedule is shown in Figure 2, and images depicting ISLT training are shown in Figure 3. The baseline (T0) session was held on the Friday afternoon of Ground Week. This session included questionnaires and balance tests (described below). Pre‐ISLT (T1) occurred the morning of ISLT (Tuesday ~0600) and included a fasting blood draw and questionnaire completion. The 4 h post‐ISLT (T2) occurred approximately 4 h after ISLT (Tuesday ~1600), and included a blood draw, balance tests, and questionnaire completion. The 18 h post‐ISLT (T3) occurred the morning after ISLT (Wednesday ~0600) and included a fasting blood draw and questionnaire completion.

### 2.3 KME/placebo intervention

D‐beta hydroxybutyrate was used for KME supplementation and was supplied directly by the manufacturer as deltaG Tactical Ketones (TdeltaS Global, Inc., Orlando, FL, USA) in bulk (1‐L bottles). The placebo beverage was a commercially prepared, taste‐matched control drink purchased from HVMN (38 Mason St., 3rd Floor, San Francisco, CA, USA) during 2019–2020 and stored according to manufacturer guidance prior to use in the present study. The placebo product was designed by the manufacturer to match the sensory characteristics of the ketone monoester beverage and contained water with noncaloric flavoring and bittering agents. Detailed proprietary formulation information and individual ingredient catalogue numbers were not available from the manufacturer at the time of the present analyses. The placebo beverage contained no ketone ingredients or caloric substrates.

To ensure appropriate dosing across participants in a timely manner, KME/PLA was administered using a tiered body weight dosing protocol. Participants and investigators were blinded to which drink was KME and PLA. Participants randomized to PLA received an equal volume of taste matched drink. All participants were categorized into one of three body weight categories based on self‐reported weight: light <72.5 kg receiving 27 g KME; medium 72.5 kg–90.7 kg receiving 35 g KME; and heavy >90.7 kg receiving 42 g KME prior to the start of ISLT training. To maintain therapeutic ketosis throughout training, participants received a 1/3‐dose booster of KME or PLA approximately 1.5 h after the initial dose (light 9 g; medium 11.7 g; heavy 14 g KME). This dosing scheme was developed and validated in two independent IRB‐approved pilot studies. In the validation study, subjects were dosed with KME over multiple days in conjunction with military relevant diet and activity while wearing a continuous ketone monitor (Lingo, Abbott). Therapeutic ketosis (>0.5 mmol/L in interstitial fluid) was sustained for 307 ± 79 min and high‐level ketosis (>2.0 mmol/L in interstitial fluid) for 188 ± 55 min in the validation study (Miyatsu et al., 2025). The protocol was therefore designed to induce high‐level ketosis during their ISLT exercise and maintain therapeutic ketosis for several hours afterwards. A finger prick testing from a randomly selected subset of participants was used to assess ketone levels in study participants during ISLT training.

### 2.4 Determination of headstrike

We obtained the instructor scoring sheet for each jump of each participant's ISLT training. To pass the ISLT exercise, students are given a maximum of 20 attempts to complete 8 successful jumps. Failed attempts are numerically scored to reflect different technical or landing failures. We collected the total number of jumps as well as the number of jumps that were scored “6” and/or “8” which correspond to whiplash and head making impact with the ground, respectively. Participants who had at least one “6” or “8” were categorized as a headstriker (HS) and those who did not were categorized as a non‐headstriker (NHS). Some participants experienced both whiplash and helmet‐to‐ground contact across one or more landing attempts; however, preliminary sensitivity analyses comparing alternative HS categorizations (e.g., separating whiplash‐only, ground‐impact–only, and combined events) did not improve sensitivity for the primary cognitive (ANAM‐4) or balance (SWAY) outcomes. Accordingly, whiplash and helmet‐to‐ground contact events were combined into a single HS category for all analyses. We also obtained information on whether a given participant completed the eight required jumps on the ISLT day and thus “passed” the ISLT exercise. Whether a given participant passed the ISLT exercise on the ISLT day was used as a target variable in multimodal predictive modeling.

### 2.5 Cognitive, behavioral, and lifestyle phenotyping

#### 2.5.1 Questionnaires

To characterize participants and collect potential moderators of ISLT exercise performance and KME response, we collected information on health history, diet, and exercise through questionnaires. Questionnaire items were grouped into three domains: (1) demographic and medical history (e.g., age, sex, prior head injuries, and medications), (2) habitual exercise and training practices (frequency, type, and intensity), and (3) dietary intake and supplement use (frequency of carbohydrate‐rich and fat‐rich foods, use of ergogenic aids). These data were used in multimodal predictive modeling.

#### 2.5.2 Fatigue and sleepiness

We used the Samn‐Perelli Fatigue Scale (Petrilli et al., 2006) to measure subjective fatigue at each data collection session. Samn‐Perelli is a seven‐point scale from “1”: “fully alert, wide awake” to “7”: “completely exhausted, unable to function efficiently.” We also collected perceptions of sleepiness using the Karolinska Sleepiness Scale (KSS) (Åkerstedt & Gillberg, 1990) to assess subjective momentary sleepiness at each data collection session. KSS is a nine‐point scale from “1”: “Extremely alert” to “9”: “Very sleepy, fighting sleep.” Both of these questionnaires were collected at Baseline, T1, T2, and T3.

#### 2.5.3 Automated neuropsychological assessment metric—Version 4, military expanded (ANAM‐4)

ANAM‐4 is a validated measurement routinely used for cognitive assessment of service members to identify neurocognitive impairments, such as those resulting from TBI (Norris et al., 2013; Vincent et al., 2017). Participants complete 10 tests from the core ANAM‐4 battery: code substitution—learning and code substitution—delayed (CDS and CDD: measure of sustained attention and working memory), go/no‐go (GNG: measure of response inhibition), matching to sample (M2S: spatial processing and visual working memory), mathematical processing (MTH: measure of basic computational skills, concentration, and working memory), procedural reaction time (PRO: information processing speed, visuomotor reaction time, and attention), spatial processing (SPD: visual skills and mental rotation), simple reaction time and simple reaction time—repeat (SRT and SRT2: reaction time), and memory search (ST6: verbal short‐term memory). ANAM‐4 was tested at Baseline and T2. ANAM showed good‐to‐excellent reliability in prior work (ICC ~0.91 for composite score) (Vincent et al., 2018).

ANAM‐4 subtests were prioritized to evaluate throughput performance metrics which incorporate both accuracy and reaction time (Jones et al., 2008), with the Go/No‐Go (GNG) test as an exception since it does not provide a throughput measure. D‐prime was used instead as it quantifies the ability to discriminate between target and nontarget stimuli based on hit rate and false alarm rate. Overall test battery mean (OTBM (Iverson et al., 2020)) was also analyzed. In all ANAM‐4 subtests, higher throughput and D‐prime values reflect more efficient information processing, faster reaction times, and greater accuracy—indicating better cognitive performance.

ANAM‐4 also included TBI Symptom Questionnaire, a self‐report inventory designed to capture common post‐concussive symptoms experienced after head impact. The questionnaire includes items assessing transient alterations in consciousness, amnesia, headache, dizziness, nausea, sensitivity to light or noise, sleep disturbance, irritability, and concentration or memory problems. Each symptom is reported dichotomously (yes/no) or by frequency/severity where applicable. The ANAM‐4 TBI Symptom Questionnaire has been widely used in military populations to aid in the identification and monitoring of concussion‐related symptoms and demonstrates good reliability and sensitivity to mild traumatic brain injury (Norris et al., 2013).

#### 2.5.4 Balance

SWAY Medical's Modified Balance Error Scoring System (mBESS) was used as a balance assessment tool designed to evaluate postural stability (Mummareddy et al., 2020). Five measurements were taken, all with eyes closed: feet together, tandem right, tandem left, single leg right, single leg left. mBESS was tested at Baseline and T2. SWAY mBESS has demonstrated moderate‐to‐good test–retest reliability (ICC ~0.76) (Mummareddy et al., 2020). For all SWAY balance metrics, higher scores indicate better postural stability and balance control, whereas lower scores reflect greater postural sway and poorer balance performance.

### 2.6 Blood collection

Blood was collected in a fasted state via venipuncture and processed for whole blood, serum, and plasma analysis. Whole blood biospecimen analysis included a complete blood count with differential and comprehensive metabolic panel performed at a clinical laboratory (BioReference Laboratories Inc). For other analyses, serum was allowed to clot at room temperature for 30 min. Plasma and serum were separated with centrifugation by spinning at 2000 g × 15 min at 4°C. Serum and plasma were transferred into 1 mL microcentrifuge tubes, frozen, and stored at −80°C.

### 2.7 Targeted TBI biomarkers, circulating inflammatory markers, and circulating hormones

Plasma and serum samples were sent overnight on dry ice to University College Dublin, Dublin, Ireland for analyses of established blood‐based markers of TBI (GFAP, UCH‐L1, Hcy, and NFL), prominent hormones and metabolism‐related proteins (TSH, T3, T4, insulin, cortisol, ApoA1, ApoB, SHGB, testosterone, estradiol, progesterone, DHEA‐sulfate, FSH, LH, prolactin) and key markers of inflammation (C3, C4, IL‐6, IL‐8, IL‐10, IL‐12 CRP, INFγ, TNFα) following manufacturer's guidelines. The targeted molecular intra‐ and inter‐assay CVs showed low variability across almost all assays and were between 2% and 5%. Only one, SHBG, had a CV above 10% and it was for the intra‐assay low concentration control (11%). Assay type, analytical platform, supplier, and catalogue numbers for all analytes are provided in Table 2.

**TABLE 2:** Assay platforms, suppliers, and catalogue (list) numbers for targeted traumatic brain injury biomarkers, circulating hormones, and inflammatory markers.

Category	Analyte	Assay type	Platform	Supplier	Catalogue (list) No.
mTBI biomarker	GFAP	Chemiluminescent immunoassay	Abbott ARCHITECT	Abbott	04W1720
	UCH‐L1	Chemiluminescent immunoassay	Abbott ARCHITECT	Abbott	04W1920
	Homocysteine (Hcy)	Chemiluminescent immunoassay	Abbott ARCHITECT	Abbott	09P2820
	Neurofilament light (NFL)	Digital immunoassay (Simoa)	Quanterix Simoa	Quanterix	104073
Hormone	Thyroid‐stimulating hormone (TSH)	Chemiluminescent immunoassay	Abbott ARCHITECT	Abbott	07P4820
	Triiodothyronine (T3), total	Chemiluminescent immunoassay	Abbott ARCHITECT	Abbott	07P9420
	Thyroxine (T4), total	Chemiluminescent immunoassay	Abbott ARCHITECT	Abbott	07P9520
	Insulin	Chemiluminescent immunoassay	Abbott ARCHITECT	Abbott	04T7520
	Cortisol	Chemiluminescent immunoassay	Abbott ARCHITECT	Abbott	08P3320
Lipoprotein	Apolipoprotein A1	Chemiluminescent immunoassay	Abbott ARCHITECT	Abbott	09P4624
	Apolipoprotein B	Chemiluminescent immunoassay	Abbott ARCHITECT	Abbott	09P4724
Sex hormone	Sex hormone–binding globulin (SHBG)	Chemiluminescent immunoassay	Abbott ARCHITECT	Abbott	09P3820
	Testosterone (2nd Gen)	Chemiluminescent immunoassay	Abbott ARCHITECT	Abbott	07P6822
	Estradiol	Chemiluminescent immunoassay	Abbott ARCHITECT	Abbott	07P5020
	Progesterone	Chemiluminescent immunoassay	Abbott ARCHITECT	Abbott	08P3620
	Dehydroepiandrosterone sulfate (DHEA‐S)	Chemiluminescent immunoassay	Abbott ARCHITECT	Abbott	09P3720
	Follicle‐stimulating hormone (FSH)	Chemiluminescent immunoassay	Abbott ARCHITECT	Abbott	07P4920
	Luteinizing hormone (LH)	Chemiluminescent immunoassay	Abbott ARCHITECT	Abbott	07P9120
	Prolactin	Chemiluminescent immunoassay	Abbott ARCHITECT	Abbott	07P6620
Inflammatory	Complement C3	Chemiluminescent immunoassay	Abbott ARCHITECT	Abbott	09P5624
	Complement C4	Chemiluminescent immunoassay	Abbott ARCHITECT	Abbott	09P5724
	Interleukin‐6 (IL‐6)	Multiplex immunoassay	Luminex	Bio‐Techne	SPCKE‐PS‐010343
	Interleukin‐8 (IL‐8)	Multiplex immunoassay	Luminex	Bio‐Techne	SPCKE‐PS‐010343
	Interleukin‐10 (IL‐10)	Multiplex immunoassay	Luminex	Bio‐Techne	SPCKE‐PS‐010343
	Interleukin‐12 (IL‐12)	Multiplex immunoassay	Luminex	Bio‐Techne	SPCKE‐PS‐010343
	C‐reactive protein (CRP)	Chemiluminescent immunoassay	Abbott ARCHITECT	Abbott	07P5620
	Interferon‐γ (IFN‐γ)	Multiplex immunoassay	Luminex	Bio‐Techne	SPCKE‐PS‐010343
	Tumor necrosis factor‐α (TNF‐α)	Multiplex immunoassay	Luminex	Bio‐Techne	SPCKE‐PS‐010343

### 2.8 Statistical analyses

We used mixed‐effect models with random intercepts to determine both three‐way (treatment: KME/PLA × HS: NHS × timepoint) and two‐way interactions (treatment × HS, treatment × timepoint, HS × timepoint) and main effects for every analysis, with the exception of the multi‐omics analyses.

### 2.9 Multimodal predictive modeling

Two complementary predictive modeling approaches were used: (1) automated criterion‐based feature selection and (2) knowledge‐based feature selection. In the automated criterion‐based feature selection approach, we used the Akaike Information Criterion (AIC) to determine whether to retain a particular feature as the predictor in a logistic regression model predicting ISLT pass/fail or HS/NHS The general formula for the AIC is as follows:

$$\mathrm{AIC}=2k-2\ln\left(L\right)$$

where *k* represents the number of parameters in the model and 𝐿 is the likelihood function of the model. It is crucial to understand that AIC values hold significance only when used to compare multiple models. For this reason, an iterative process was used where features were systematically removed and reintroduced, allowing observation into how these changes impacted the AIC value. For knowledge‐guided feature selection, features were profiled to identify initial trends and examine distributions, correlations, and missing values. Through an iterative selection process guided by domain knowledge and correlation analysis, features were down‐selected to only those that improved classification accuracy. Features included participant characteristics, SWAY balance tests, and ANAM cognitive reaction metrics. The final feature set includes: participant characteristics: BMI and age; SWAY: feet together eyes closed; ANAM: Simple Reaction Time.

The modeling approach employed a range of classification techniques, beginning with Naive Bayes and logistic regression as baselines, and progressing through support vector machines (SVM), Gaussian Process, gradient boosting, and Random Forest models. The best results were obtained using Random Forest. Given the imbalanced nature of the data, with fewer instances of high‐risk participants, class weighting was applied to improve classification balance. Model hyperparameters were tuned using cross‐validation with the F1‐score and Brier score, as these metrics are better suited for evaluating performance on imbalanced datasets. Results indicated that F1‐optimized models outperformed those optimized for Brier score, so we selected the F1‐optimized model for final evaluation.

To address the imbalances in some of the data (e.g., HS vs. NHS, ISLT pass vs. fail) and ensure a fair representation in the modeling process, a strategy of random sampling was implemented during the 80–20 cross‐validation process. An equal number of participants from each group were selected to create a balanced dataset. The resulting dataset was then split into training and testing subsets, with 80% of the data used for training the model and 20% reserved for testing its performance. The multivariable logistic regression model was trained on the 80% training subset and subsequently evaluated on the hold‐out 20% test subset. Model performance was assessed using accuracy as the primary metric.

## 3 RESULTS

### 3.1 Participant breakdown

Across our 8 independent cohorts, 354 active‐duty service members were enrolled and randomized to KME/PLA with 327 successfully completing ISLT [PLA = 163, 147 M/16F; KME (*n* = 164, 146 M/18F)]. General demographics and anthropometrics for these individuals are shown in Table 1. 311 (95%) of these participants successfully completed ISLT (8 successful jumps within 20 attempts). 11 of the 327 participants were removed from ISLT by the cadre instructors for reasons other than significant head impacts. Five of the 327 were removed from ISLT by cadre due to significant head impacts.

### 3.2 Headstrike determination

We elected to use cadre's scoring of the ISLT performance as the variable to quantify the head impact. Cadre scoring of the 327 that finished ISLT resulted in 105 (32.1%) with a scored headstrike, with 55 (16.8%) receiving at least one score of “6” (whiplash) and 50 (15.3%) with at least one score of “8” (helmet‐to‐ground impact). Figure 3 shows the frequency histogram depicting the distribution of the number of head injuries experienced by participants broken down by the treatment group.

### 3.3 Analytic treatment of headstrikes

Preliminary analyses compared outcomes for participants experiencing whiplash versus helmet‐to‐ground impacts and found that the effects on cognitive and balance performance were similar. Accordingly, these subtypes were combined into a single HS category to maximize statistical power. We also examined headstrike frequency by treating HS as a continuous variable and by categorizing participants with multiple HS separately. Neither approach improved sensitivity or interpretability of the analyses, so we elected to dichotomize the HS variable as HS versus no‐HS in all subsequent analyses.

### 3.4 Symptoms based on headstrikes

The responses for the ANAM‐4 TBI symptom questionnaire were extremely sparse. Only three of the 43 items received measurable responses, and none differed significantly between the HS versus no‐HS groups (concussion symptoms: no‐HS 3.9% (10/255) vs. HS 8.8% (6/68), OR 0.41 [0.15, 1.14], *p* = 0.115; feeling dazed: no‐HS 7.1% (18/255) vs. HS 11.8% (8/68), OR 0.55 [0.23, 1.31], *p* = 0.213; headache: no‐HS 9.4% (24/255) vs. HS 10.3% (7/68), OR 0.87 [0.37, 2.06], *p* = 0.818). The five participants who were removed from ISLT by cadre due to significant head impacts did not complete the questionnaire. Given the low response rate and lack of group differences, sub‐analyses based on symptom variables were not conducted.

### 3.5 Fatigue and alertness (T1, T2, and T3)

**FIGURE 4:**
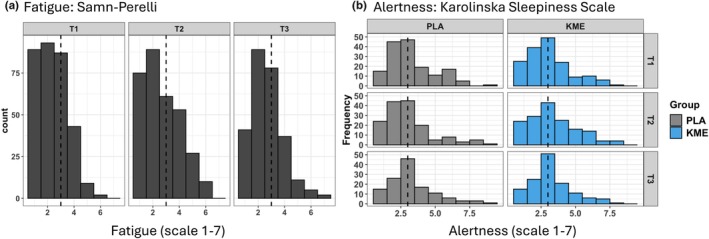
The distribution of participants' fatigue and sleepiness rating across timepoint. Baseline (T0): Friday, two days prior to ISLT; T2: 4–6 h post ISLT.

There were no group × time interaction effects noted for fatigue using the Samn‐Perelli but there was a significant main effect of time (*p* < 0.001) as shown by a shift in the frequency distribution of fatigue ratings in Figure 4 below, panel a. There was a significant treatment by time interaction in the KSS (*p* = 0.020). However, when visualizing the frequency distribution (Figure 4, panel b), there was no shift in the frequency of reporting of higher scores on the KSS in KME vs. PLA.

### 3.6 KME confirmation

Fingerstick blood ketone levels were measured 30–60 min after KME/PLA administration from 2 to 4 randomly chosen participants from each of the eight cohorts. It showed successful administration as PLA averaged 0.28 mmol/L [range: 0.1–0.3] and KME an average level of 2.01 mmol/L [range: 0.8–7.4]. Due to the fast‐paced training environment and the large number of participants undergoing ISLT simultaneously (up to >50 individuals at a time), it was not feasible to obtain BHB measurements from all participants at every time point. Therefore, sampling was performed in a representative subset to minimize disruption to training.

### 3.7 Cognitive and balance performance (baseline and T2)

#### 3.7.1 ANAM‐4

**FIGURE 5:**
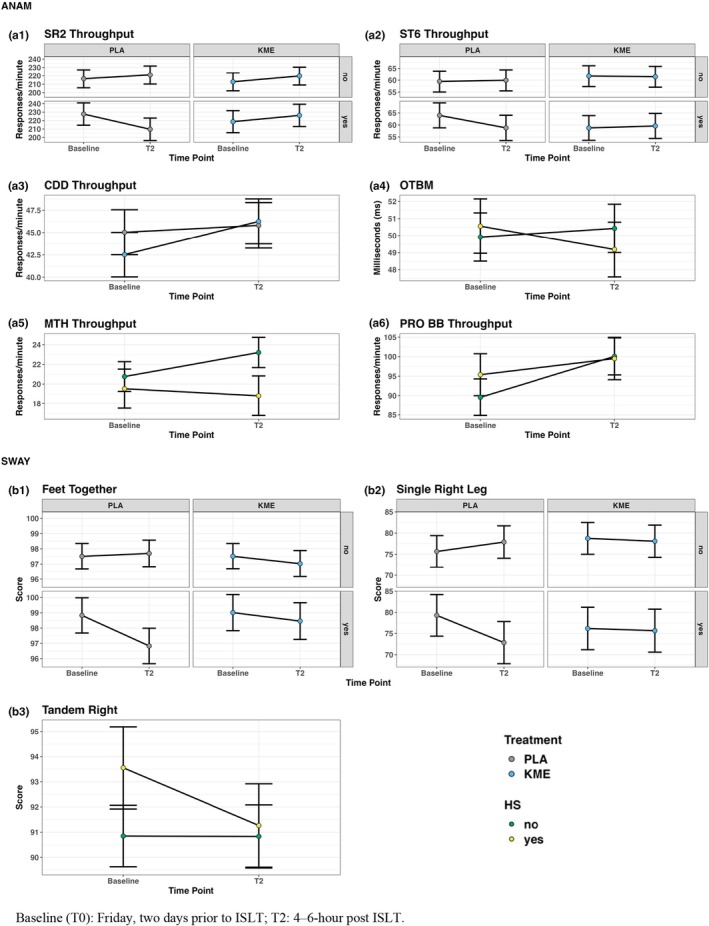
Model‐estimated group means for the significant two‐way and three‐way interaction results on ANAM‐4 and SWAY analysis. The error bars denote the model‐estimated 95% CI.

One of the 11 comparisons had a significant three‐way HS × timepoint × treatment interaction: Simple Reaction Time – Repeat (SRT2; *p* = 0.024). Post‐test comparisons showed PLA + HS showed a decrease (*p* = 0.002) not seen in the other three conditions. Go/No‐Go D‐prime showed low probability of there being no three‐way interaction (*p* = 0.066). Figure 5 depicts all the significant three‐way and two‐way interaction patterns from the 11 comparisons.

Regarding the two‐way interactions, 3 out of the 11 comparisons (i.e., 10 subtests plus OTBM) had a significant interaction. OTBM had a significant HS × timepoint interaction (*p* = 0.025) with post‐testing showing OTBM decreases in HS (*p* = 0.043) but not NHS (*p* = 0.303). For specific subtests, Mathematical Processing had a significant HS × Time interaction (*p* = 0.042) with improved Mathematical Processing throughput in NHS at T2 compared to Baseline (*p* = 0.009) whereas it was unchanged in the HS individuals (*p* = 0.564). Procedural Reaction Time also had a HS × Time interaction (*p* = 0.033) with NHS improvement in Procedural Reaction Time throughput from Baseline (*p* < 0.001) while no improvement was noted in HS (*p* = 0.085).

#### 3.7.2 SWAY

Single Leg Right had a significant three‐way interaction (*p* = 0.014; Figure 5 b2) as performance decreased from baseline to T2 among PLA HS individuals (*p* = 0.001) but not in others (KME HS: *p* = 0.807, PLA NHS: *p* = 0.134, KME NHS: *p* = 0.645). Feet Together did not meet a prior alpha, although there was a low probability that there was no three‐way interaction (*p* = 0.081; Figure 5 b1). For two‐way interactions focusing on HS × Time interactions, Tandem Right (*p* = 0.042; Figure 5 b3) performance decreased in HS (*p* = 0.012) compared to baseline where it was unchanged in NHS (*p* = 0.982).

### 3.8 mTBI targeted plasma biomarkers (T1, T2, and T3)

#### 3.8.1 mTBI Biomarkers for full cohort (HS/NHS)

**FIGURE 6:**
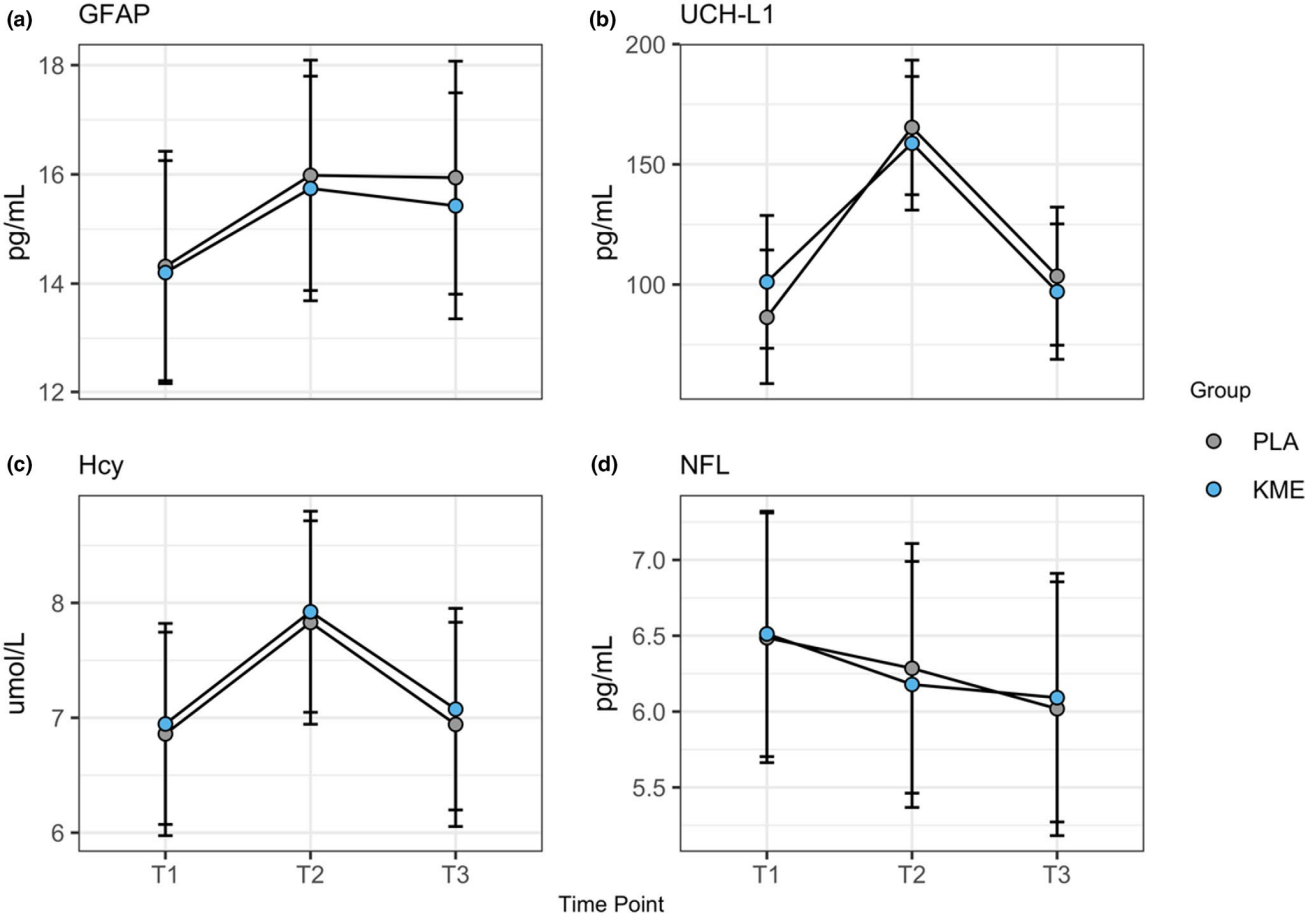
Model‐estimated group means for the four mTBI biomarkers. The error bars denote the model‐estimated 95% CI. GFAP, glial fibrillary acidic protein); UCH‐L1, ubiquitin C‐terminal hydrolase L1; Hcy, homocysteine; NFL, neurofilament light. T1, Early morning fasted blood draw prior to ISLT; T2: 4–6‐h post ISLT blood draw; T3: Early morning fasted blood draw 18–20 h post ISLT.

A total of 317 participants were included in the full analysis (HS/NHS). The first analysis was only looking at the HS × timepoint interaction to determine whether HS influenced changes in mTBI biomarker levels across timepoints. There were no interactions for GFAP (*p* = 0.355), UCH‐L1 (*p* = 0.343), Hcy (*p* = 0.788), or NFL (*p* = 0.832). There were main effects of time for UCH‐L1 (*p* < 0.001) and Hcy (*p* < 0.001). and trends for GFAP (*p* = 0.100). There was not a main effect of time for NFL (*p* = 0.298). This is shown in Figure 6.

#### 3.8.2 mTBI biomarkers in HS

Subset analyses examining treatment (KME/PLA) × timepoint interactions were next completed to evaluate the primary aim of determining whether KME had protective effects on mTBI biomarker levels. There were a total of 109 with mTBI biomarker data and who sustained a HS with 54 in PLA and 55 in KME. There were no group by time interactions for GFAP (*p* = 0.831), UCH‐L1 (*p* = 0.201), Hcy (*p* = 0.967), or NFL (*p* = 0.851) (Figure 6). However, there were main effects of time for all four mTBI biomarkers (UCH‐L1: *p* < 0.001, GFAP: *p* < 0.001, Hcy: *p* < 0.001, and NFL: *p* = 0.027). UCH‐L1 was increased at T2 versus T1/T3 (*p* < 0.001). GFAP was elevated at T2/T3 (*p* < 0.001). Hcy followed a similar pattern as UCH‐L1 with T2 greater than T1/T3 (*p* < 0.001). NFL was greater at T3 compared to T1 (*p* = 0.009).

### 3.9 Hormones and cytokines (T1 and T3)

**FIGURE 7:**
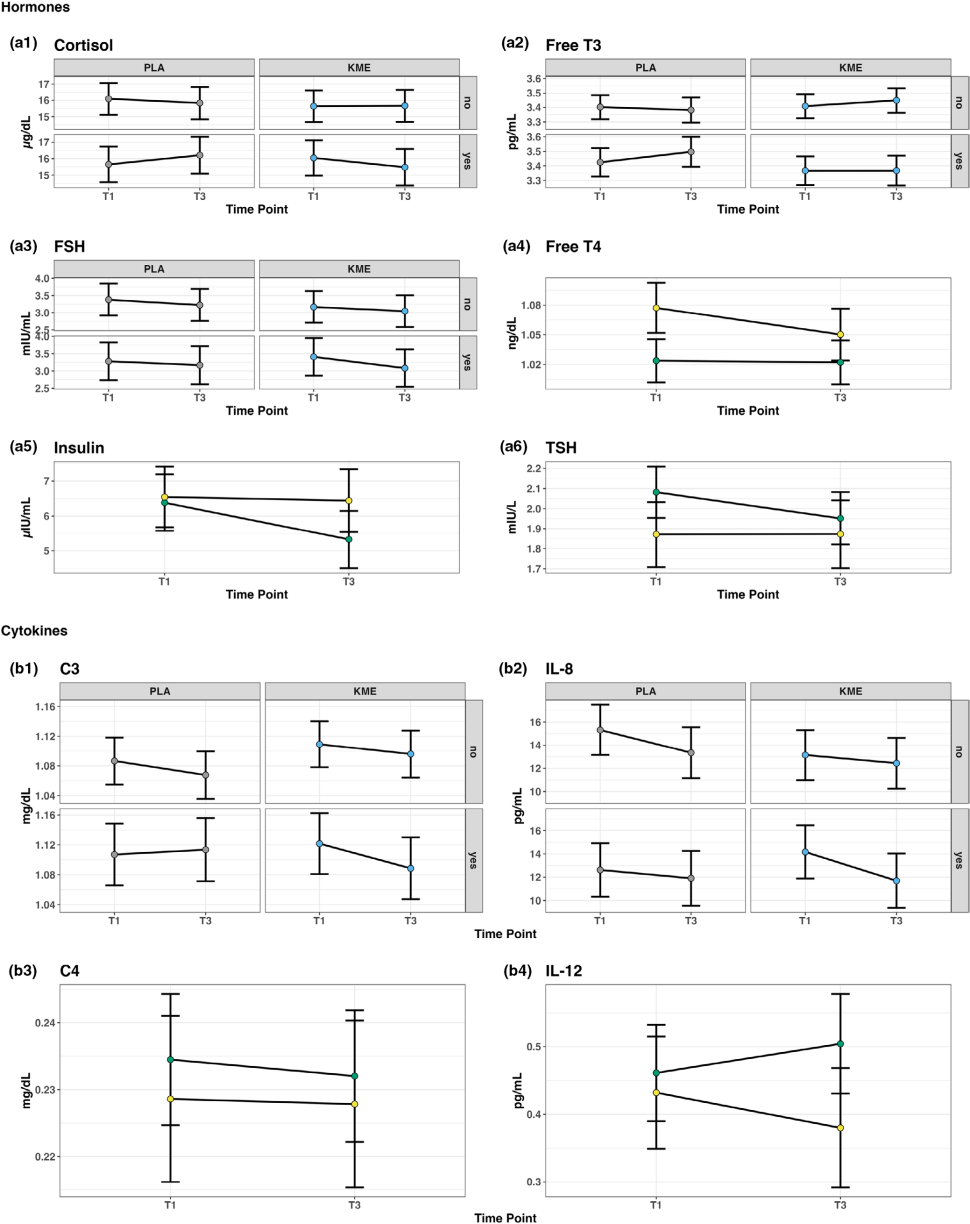
Model‐estimated group means for the hormones and cytokines analysis. The error bars denote the model‐estimated 95% CI.

Figure 7 shows all the significant two‐way and three‐way interactions from the hormones and cytokines analysis. There were no three‐way interactions for TSH, total T3 or total T4 (*p* > 0.05). There was a two‐way HS × time interaction with a decrease in TSH in NHS (*p* = 0.003) whereas there was no change in HS from T1 to T3 (*p* = 0.980). Free T4 had a two‐way interaction between HS and timepoint (*p* = 0.012) where it was decreased from T1 to T3 in HS (*p* = 0.001) but remained unchanged at T3 versus T1 in NHS (*p* = 0.792). There was a significant three‐way interaction for free T3 (*p* = 0.041); however, there were no statistical differences following post‐testing from T1 to T3: PLA NHS (*p* = 0.437), KME NHS (*p* = 0.135), KME HS (*p* = 0.997), or PLA HS (*p* = 0.056). Insulin showed a significant HS × time interaction (*p* = 0.016) but no three‐way interaction (*p* = 0.514). There was a decrease in insulin from T1 to T3 in the NHS group (*p* < 0.001) that did not occur in the HS group (*p* = 0.749). There was a significant three‐way interaction for cortisol (*p* = 0.043); however, when the simple main effects were examined there were no changes from T1 to T3 in HS (KME: *p* = 0.156; PLA: *p* = 0.168) or NHS (KME: *p* = 0.915; PLA: *p* = 0.377). There were no significant interactions for ApoA1 or ApoB (all interaction *p* > 0.100).

#### 3.9.1 Sex hormones

There were no significant interactions for SHBG or the key hormones it regulates: testosterone (all interactions *p* > 0.270), estradiol (all interactions *p* > 0.280), or DHEA‐Sulfate (all interactions *p* > 0.383). There was a significant three‐way interaction for FSH (*p* = 0.047) with decreases in KME HS (*p* < 0.001), KME NHS (*p* = 0.018), and PLA NHS (*p* = 0.003), but did not decrease statistically in PLA HS (*p* = 0.122). There was a low probability that the response in prolactin from T1 to T3 was not impacted by HS versus No HS (HS × timepoint: *p* = 0.055). There were no significant interactions for LH (all interactions *p* > 0.202) or progesterone (all interactions *p* > 0.326).

#### 3.9.2 Cytokines and complement proteins

There were significant three‐way interactions for C3 (*p* = 0.010) and IL‐8 (*p* = 0.024). For C3 the interaction was primarily driven by a lack of decrease in PLA HS (*p* = 0.523), that was seen in PLA NHS (*p* = 0.010) and KME HS (*p* < 0.001). The three‐way interaction for IL‐8 is potentially driven by IL‐8 being elevated at T1 in PLA NHS and KME HS at T1. This is evidenced by the finding that PLA NHS was significantly higher than PLA HS (*p* = 0.001) and KME NHS (*p* = 0.002), but not different from KME HS at T1 (*p* = 0.162). IL‐8 decreased in both the PLA NHS (*p* < 0.001) and KME HS (*p* = 0.001), but not in KME NHS (*p* = 0.205) or PLA HS (*p* = 0.351). All groups had no differences in mean values at T3, suggesting that the change in PLA NHS and KME HS were regression to the mean with physiological relevance questioned. There were no other three‐way interactions for inflammatory cytokines (CRP, INFγ, IL‐6, IL‐10, and TNFα). For the lower order interactions there were two: HS × timepoint for IL‐12 (*p* = 0.048) and a treatment × timepoint for C4 (*p* = 0.036). Post‐tests for IL‐12 showed a strong increase in KME NHS (*p* = 0.007) while there were no changes for any other group (KME HS: *p* = 0.553; PLA NHS: *p* = 0.630; PLA HS: *p* = 0.195). For C4, post‐tests demonstrated a small decrease in C4 in KME from T1 to T3 (*p* = 0.006) but not in PLA (*p* = 0.790). There were no differences between groups at T1 (*p* = 0.224) or T3 (*p* = 0.488).

### 3.10 Predictive modeling

#### 3.10.1 Automated criterion‐based feature selection models

There were three classes of baseline variables that we considered in automated criterion‐based feature selection models predicting HS/NHS and ISLT pass/fail: blood‐based targeted biomarkers, cognitive and balance performance variables (ANAM and SWAY), and lifestyle variables (e.g., diet and exercise). Untargeted, discovery‐based transcriptomics and proteomics were not included in modeling due to sample size limitations (discovery platforms were run on a subset of 66 participants). Baseline biomarkers did not exhibit meaningful relationship with the dependent variables. Table 3 shows the list of predictor variables that improved model's AIC criterion and thus contributed to better prediction along with their *b* estimate, standard error, and *z* and *p* value in the regression model used in the final prediction and validation. In the model predicting the HS/NHS (top panel), Feet Together (SWAY), Single Leg Right (SWAY), and median RT for correct trials on Mathematical Processing (ANAM) emerged with significant or marginally significant (*p* < 0.100) *p* values. The smaller *p* values associated with these features suggest that baseline cognitive and balance performance hold significant predictive capability for differentiating between NHS/HS statuses. The predictive accuracy of this model using the validation approach with the balanced dataset was 71.58% with average precision of 68.67%, average recall of 74.62%, and average F1‐score of 71.52%.

**TABLE 3:** Regression table with accuracy summarizing the models predicting HS/NHS and ISLT pass/fail from cognitive/ balance and lifestyle variables.

DV and accuracy	Predictor variable	Estimate	Std. error	z value	Pr(>|z|)
HS/NHS Accuracy: 71.58%	(Intercept)	−2.24E+01	1.07E+01	−2.096	0.03608*
	Tandem Right (SWAY)	1.19E−01	4.19E−02	2.843	0.00447*
	Feet Together (SWAY)	1.39E−01	1.07E−01	1.298	0.19415
	Single Leg Right (SWAY)	−3.16E−02	1.65E−02	−1.92	0.05489
	Code Substitution Delayed (ANAM‐4)	−2.28E−03	1.27E−03	−1.788	0.07377
	Matching to Sample (ANAM‐4)	9.74E−04	6.97E−04	1.397	0.16243
	Mathematical Processing (ANAM‐4)	4.42E−04	2.43E−04	1.818	0.06905
	High intensity endurance training per week (min)	−8.27E−03	5.75E−03	−1.438	0.15049
	Low intensity endurance training per week (min)	−5.16E‐01	5.08E+01	−0.01	0.9919
ISLT (Pass/Fail) Accuracy: 74.50%	(Intercept)	6.76E+00	1.68E+01	0.402	0.68794
	Feet Together (SWAY)	−4.09E−01	1.69E−01	−2.428	0.01518*
	Code Substitution Delayed (ANAM‐4)	6.68E−03	3.02E−03	2.21	0.02708*
	Code Substitution Learning (ANAM‐4)	1.04E−02	4.05E−03	2.569	0.01022*
	Mathematical Processing (ANAM‐4)	−2.21E−03	9.85E−04	−2.24	0.025*
	Procedural Reaction Time (ANAM−4)	2.42E−02	8.84E−03	2.735	0.00624*
	Spatial Processing (ANAM‐4)	−4.55E−03	1.63E−03	−2.791	0.00526*
	Simple Reaction Time (ANAM‐4)	6.44E−03	2.66E−03	2.424	0.01537*
	Memory Search (ANAM‐4)	−8.82E−03	4.56E−03	−1.936	0.05289
	High intensity endurance training per week (min)	1.11E−02	8.15E−03	1.363	0.1734
	Mod intensity endurance training per week (min)	1.43E−02	6.05E−03	2.359	0.01834*
	Low intensity endurance training per week (min)	6.45E−01	6.63E+01	0.01	0.99234
	Height	1.74E−01	7.47E−02	2.335	0.01955*
	Weight	−1.26E‐01	5.77E−02	−2.178	0.02937*
	Ketogenic diet	1.50E+01	8.57E+00	1.75	0.08021
	Intermittent fasting	−1.30E+01	8.45E+00	−1.542	0.1231

* *p* < 0.05.

In the model predicting the ISLT pass/fail (bottom panel), Feet Together (SWAY), median RT for correct trials on various ANAM variables, such as Procedural Reaction Time, Code Substitution ‐Learning and ‐Delayed, Spatial Processing, Memory Search, duration of moderate endurance exercise per week, height, and keto diet emerged with significant or marginally significant (*p* < 0.100) *p* values. The predictive accuracy of this model using the validation approach with the balanced dataset was 74.50% with average precision of 77.10%, average recall of 72.80%, and average F1‐score of 74.89%.

#### 3.10.2 Knowledge‐guided feature selection models

The predictive modeling effort with the knowledge‐guided feature selection approach focused on developing Random Forest models on HS risk prediction. The models predicting BAC training pass/fail were unable to capture any meaningful patterns. In developing a Random Forest model that predicts HS, a clear pattern of improvement in predictive accuracy was observed. In the first step, demographic and lifestyle variables were used, and higher BMI and age emerged as significant predictors. The accuracy of the base model using these two variables as predictors was 46%. In the second step, SWAY balance performance variables were considered. Feet Together emerged as a significant predictor, and adding this variable improved the model's accuracy from 46% to 61%. In the third and final step, ANAM‐4 cognitive performance variables were added, and Simple Reaction Time emerged as a significant predictor. Adding this variable improved the model's accuracy from 61% to 74%. The model evaluation metrics for each step are reported below in Table 4.

**TABLE 4:** The accuracy metrics of the three Random Forest models with varying sets of predictors predicting HS/NHS.

Predictor	Precision		Recall		F1‐score		Support		Overall
	High	Low	High	Low	High	Low	High	Low	Acc.
BMI + age only	0.20	0.58	0.19	0.60	0.19	0.59	16	30	45%
+ feet together eyes closed	0.40	0.67	0.25	0.80	0.31	0.73	16	30	61%
+ simple reaction time	0.64	0.78	0.56	0.83	0.60	0.80	16	30	74%

## 4 DISCUSSION

This study primarily sought to determine HS incidence, evaluate whether HS was associated with cognitive and balance performance changes, and test whether prophylactic KME supplementation could mitigate these decrements. Secondary objectives examined targeted blood biomarkers, while exploratory analyses included predictive modeling to generate hypotheses regarding molecular signatures and risk factors for HS. Our data show that 33% of ISLT trainees experienced at least one head strike event (i.e., whiplash or helmet making audible contact with the ground). HS was associated with modest changes in a small number of measurements of cognitive and balance performance; these individuals did not have either targeted protein or discovery‐based biomarker signatures indicative of mTBI. KME demonstrated limited protective effects in cognitive and balance performance variables altered by HS, but understanding the physiological relevance and potential mechanism is hindered by the lack of change in blood‐based mTBI biomarkers.

### 4.1 Headstrike prevalence and severity

Of the 386 participants enrolled and 354 randomized across eight study cohorts investigated June 2022 until November 2023, 327 participants completed the study. We leveraged the cadre score as the “ground truth” for HS, which is defined as whiplash (score of “6”) or clear (audible or visible) helmet collision with the ground (score of “8”). Cadre instructors are well‐trained and experienced in grading ISLT performance, but scoring is not quantitative. Our observations that there was no clear pattern of pass/fail and general scoring bias across cohorts suggest variability in cadre scoring between instructors was minimal. The use of the cadre scoring, while similar across cohorts, could also overrepresent the number of significant HS. Whiplash and observable helmet contact with the ground can certainly induce detectable force that gets transmitted through the helmet. However, our complete cohort had few withdrawals due to severe enough collisions that resulted in concussion‐like symptoms.

This study focused on acute effects of HS during airborne training, and we did not assess long‐term or chronic neurological sequelae. While repetitive head impacts have been linked to persistent neurocognitive changes in other contact and military training contexts, the potential for chronic effects associated specifically with airborne landings remains unclear and warrants future longitudinal investigation.

### 4.2 Cognitive and balance performance

In analyzing ANAM‐4 and SWAY data, we focused on answering two critical questions: (1) Did ISLT produce HS‐dependent decline in cognitive and balance performance, and (2) whether KME supplementation attenuated this decline. Regarding the first question, four out of the 11 comparisons (i.e., 10 ANAM‐4 subtests plus overall test battery mean: OTBM) in ANAM variables had a 2‐ or 3‐way interaction in the hypothesized direction indicating HS‐dependent declines in overall (OTBM) and subtests (Mathematical Processing, Procedural Reaction Time, and Simple Reaction Time – Repeat). Similarly, two out of five comparisons in SWAY variables (Single Leg Right, Feet Together) showed a significant decline or a strong trend towards decline. While the marginally significant trend in Feet Together data should be interpreted with caution as participants' performance in this test approached ceiling, these results indicate that there was a modest signature of HS‐dependent decline in cognitive and balance performance.

KME supplementation showed limited efficacy in attenuating this cognitive and balance performance decline, as only one out of the 11 comparisons (Simple Reaction Time – Repeat) had a significant three‐way interaction. Thus, the current data provide insufficient evidence to conclude overall efficacy. Similarly, only one out of five comparisons in SWAY variables (Single Leg Right) showed a significant three‐way interaction. Thus, these results also provide limited support for KME's efficacy to protect balance performance against HS.

### 4.3 Targeted blood‐based biomarkers

Our targeted molecular biomarker data strongly suggests the observed HS sustained during BAC ISLT were not severe enough to induce detectable mTBI. We used both FDA‐approved markers (GFAP and UCH‐L1) and well‐established markers of neuronal damage (homocysteine and NFL). Circulating values of ≥30 pg/mL for GFAP and ≥360 pg/mL for UCH‐L1 indicate possible moderate to severe TBI (Bazarian et al., 2021) and would result in referral for CT scan in clinical settings, while a recent meta‐analysis showed potential clinical thresholds for mTBI are widely varied due to platform and assay (Amoo et al., 2022). In our participants, mean GFAP ranged from 12.5 to 18.09 pg/mL, far below any indication of TBI and no difference observed between HS and NHS. This was similar for UCL‐L1, as the confidence interval ranged from 58.4 to 193.4 pg/mL, well below the clinical threshold of 360 pg/mL. Our biospecimen collection schedule was largely identical across cohorts as demonstrated by time‐based changes we observed showing circadian and/or diet induced changes for these factors. But it is clear there were not any detectable differences between those that received a HS and those that did not. Furthermore, a recent selective review shows blood‐based profiling is not indicative of mTBI from concussive or sub‐concussive forces and cannot repeatedly discern healthy controls and concussed individuals (Hier et al., 2021). Lastly, we did not perform clinical testing for traumatic brain injury (e.g., Glascow Coma Scale) so there is no clear way to link persons with one or multiple scored HS to a clinically relevant outcome.

### 4.4 Hormones and cytokines

We did note some unique interaction effects across the groups and timepoints in both our complex three‐way model, as well as the reduced complexity two‐way models. The practical implication from these interaction effects is not clear, as the magnitude of change was relatively minimal and there was still substantial overlap of the predicted confidence intervals. However, similar to the direct TBI markers, we observed changes reflective of circadian rhythm, activity or diet as the T2 timepoint had the greatest number of statistically different concentrations of hormones and cytokines. T1 and T3 collections were fasted morning, typically taken between 0600 and 0700 across all cohorts, with T2 taken between 1600 and 1700. Physical activity throughout the day as well as T2 being closer to their mid‐day meal is likely the main driver of these outcomes. As no HS was severe enough to create a primary injury and the resultant secondary inflammatory and stress response, it is not surprising there is no clear effect of HS on these factors.

### 4.5 Multiomics

We also conducted exploratory transcriptomic and proteomic analyses but did not identify reproducible molecular signatures of HS or KME supplementation. While small sets of differentially expressed transcripts and proteins were observed at isolated timepoints, these changes were inconsistent and often attributable to time‐of‐day variation. These findings align with the targeted biomarker data, reinforcing the conclusion that HS events in this training context did not produce detectable molecular signatures of mTBI. The complete exploratory multi‐omics datasets and associated analyses have been deposited as a permanent public record in Figshare (DOI: 10.6084/m9.figshare.31021216); these data are provided to support transparency and future hypothesis generation and were not subjected to formal peer review as part of the primary manuscript.

### 4.6 Predictive modeling

Predictive modeling suggested that baseline cognitive and balance performance, along with demographic variables such as age and body mass index (BMI), were modestly associated with headstrike (HS) risk during ISLT. Across complementary modeling approaches, classification accuracy was moderate (≈71%–74%), indicating that pretraining neurocognitive and postural stability measures contain informative but incomplete signal for HS risk stratification. Contributing features consistently included measures of reaction time and balance, supporting the premise that baseline sensorimotor function may influence landing performance and susceptibility to HS. However, limited sensitivity for identifying higher‐risk individuals and class imbalance constrained predictive utility, underscoring the need for larger datasets, improved biomechanical characterization, and more precise phenotyping to achieve operationally actionable prediction.

### 4.7 Limitations

A major limitation of this study is the absence of usable head kinematics data during ISLT landings. Although in‐helmet sensors were deployed, signal quality was insufficient for analysis, underscoring the difficulty of capturing reliable biomechanical data in large‐scale, field‐based military training environments. As a result, we were unable to quantify linear and rotational head accelerations associated with individual landings, which limits the ability to relate impact magnitude or direction to acute cognitive, balance, or biomarker outcomes. Future studies should incorporate validated wearable sensor systems to quantify impact magnitude and direction, which would strengthen the interpretation of head strike events.

An additional limitation is the absence of a nonimpact time control condition. While repeated pre‐ and post‐ISLT assessments were used to account for within‐day variability, the inclusion of a matched control condition without head impact exposure would have strengthened causal inference by better isolating the effects of head strike from time‐of‐day, fatigue, or training‐related influences. The operational constraints of a high‐throughput military training environment precluded the inclusion of such a control group; however, future studies incorporating parallel non‐impact training days or matched non‐airborne cohorts would improve experimental control.

Another limitation concerns the KME dosing strategy. Due to logistical constraints of a large‐scale military training environment, it was not feasible to confirm therapeutic ketosis in all participants, and BHB was measured only in a subset. Although dosing was administered shortly before ISLT, unanticipated wait times associated with the unpredictable pace of training likely resulted in variable ketone levels at the time of task engagement. This uncertainty makes it difficult to determine whether null or limited findings reflect a lack of biological efficacy or suboptimal timing of ketosis. Future studies could benefit from the use of continuous ketone monitoring to better characterize real‐time ketone dynamics during operational tasks. In addition, while our study employed an acute dosing strategy, it is possible that chronic supplementation may sustain ketosis more effectively and yield greater protective benefits, an important avenue for future investigation.

## 5 CONCLUSIONS

The current study aimed to determine the prevalence and consequences of HS during US Army BAC training and assess the potential efficacy of prophylactic ketone monoester (KME) supplementation in mitigating secondary outcomes of mild traumatic brain injury (mTBI). Through a combination of cognitive, balance, blood‐based biomarkers, and multimodal analyses, our findings provide new insights into ISLT training HS incidence and associated performance changes. This study confirmed that HS during ISLT training produced measurable cognitive and balance deficits, even in the absence of biomarker levels suggestive of mTBI. While KME supplementation showed limited prophylactic efficacy on performance outcomes, the findings pave the way for further research into neuroprotective strategies and predictive tools for mitigating the risks associated with repetitive head impacts in military personnel. Future work should focus on refining detection methods, expanding phenotyping, and exploring novel interventions to safeguard warfighter performance and long‐term neurological health.

## AUTHOR CONTRIBUTIONS

T.M., J.M., and Z.A.G. contributed to methodology, investigation, formal analysis, data curation, software, validation, visualization, and writing of the original draft, as well as review and editing of the manuscript. A.M. and K.M. contributed to formal analysis, software, writing of the original draft, and manuscript review and editing. A.D. contributed to investigation and visualization. M.Y. contributed to investigation, supervision, and project administration. A.F. contributed to methodology, investigation, and manuscript review and editing. E.A., E.H., B.L., K.G.‐M., B.M., J.P., R.S., K.V.K.‐J., and P.P. contributed resources. M.M.B., T.J.B., and M.S. contributed to conceptualization, methodology, investigation, supervision, manuscript review and editing, and funding acquisition. All authors approved the final manuscript.

## FUNDING INFORMATION

This work was supported by the United States Department of Defense, United States Special Operations Command (SOCOM), under Contract No. H9240519C0016 awarded to the Florida Institute for Human & Machine Cognition. Additional support for related omics analyses was provided by the National Institutes of Health, National Cancer Institute, under Award No. P30CA033572. Abbott Diagnostics performed mTBI biomarker assays in a blinded manner as an in‐kind contribution. The Alinity I TBI test was developed in collaboration with the U.S. Army Medical Materiel Development Activity, U.S. Army Medical Research and Development Command, under Cooperative Research and Development Agreement CRADA 20‐1266‐CRA. Omics analyses were conducted by the Translational Genomics Research Institute team with support from the National Cancer Institute. Results from a separate but related validation study (Miyatsu et al., 2025) are also reported, for which Abbott Lingo provided continuous ketone monitoring devices as an in‐kind contribution.

## ETHICS STATEMENT

All procedures involving human participants were reviewed and approved by the Institutional Review Board of the Florida Institute for Human and Machine Cognition (IHMC; protocol #2022‐0031) and the U.S. Army Medical Research and Development Command Office of Human Research Oversight. The study was conducted in accordance with the Declaration of Helsinki and applicable institutional guidelines, and written informed consent was obtained from all participants prior to participation.

## Data Availability

The exploratory multi‐omics methods, analysis outputs, and discussion from this study have been deposited in Figshare and are publicly available at https://figshare.com/s/4f7b64b39fbf7e0e5f2d. (DOI: 10.6084/m9.figshare.31021216). They are provided as a permanent public record and represent exploratory analyses that were not subjected to formal peer review as part of the primary manuscript. Additional data supporting the findings of this study, including de‐identified individual‐level data not contained in the Figshare repository, are available from the corresponding author upon reasonable request and subject to institutional review board (IRB), ethical, and data‐use agreement approvals.
